# A novel biomarker TERTmRNA is applicable for early detection of hepatoma

**DOI:** 10.1186/1471-230X-10-46

**Published:** 2010-05-18

**Authors:** Norimasa Miura, Yukio Osaki, Miki Nagashima, Michimori Kohno, Kensho Yorozu, Kohei Shomori, Takamasa Kanbe, Kenji Oyama, Yukihiro Kishimoto, Shigeo Maruyama, Eijiro Noma, Yutaka Horie, Masatoshi Kudo, Seigo Sakaguchi, Yasuaki Hirooka, Hisao Ito, Hironaka Kawasaki, Junichi Hasegawa, Goshi Shiota

**Affiliations:** 1 Division of Pharmacotherapeutics, Department of Pathophysiological and Therapeutic Science, Faculty of Medicine, Tottori University, 86 Nishicho, Yonago, Tottori 683-8503, Japan; 2 Department of Gastroenterology, Osaka Red Cross Hospital, 5-30 Fudegasaki-cho, Tennouji-ku, Osaka, Osaka 543-8555, Japan; 3 Department of gastroenterology, Kinki University, 3-4-1 Kowakae, Higashi-Osaka, Osaka 577-8502, Japan; 4 Department of Gastroenterology, Matsue City Hospital, 32-1 Noshira-cho, Matsue, Shimane 690-8509, Japan; 5 Department of Internal Medicine, Shimaneken Saiseikai Gotsu General Hospital, 1551 Gotsu-cho, Gotsu, Shimane 695-8505, Japan; 6 Division of Organ Pathology, Faculty of Medicine, Tottori University, Nishicho 86, Yonago, 683-8503, Japan; 7 Internal Medicine, San-in Labor Welfare Hospital, 1-8-1 Kaikeshinden, Yonago, Tottori 683-0002, Japan; 8 Department of Gastroenterology, Fukuoka University Chikushi Hospital, 1-1-1 Zokumyoin, Chikusino, Fukuoka 818-8502, Japan; 9 Department of Pathobiological Science and Technology, School of Health Science, Faculty of Medicine, Tottori University, 86 Nishicho, Yonago, Tottori 683-8503, Japan; 10 Division of Molecular and Genetic Medicine, Department of Genetic Medicine and Regenerative Therapeutics, Tottori University School of Medicine, 86 Nishicho, Yonago, Tottori 683-8503, Japan

## Abstract

**Backgrounds:**

We previously reported a highly sensitive method for serum human telomerase reverse transcriptase (hTERT) mRNA for hepatocellular carcinoma (HCC). α-fetoprotein (AFP) and des-γ-carboxy prothrombin (DCP) are good markers for HCC. In this study, we verified the significance of hTERTmRNA in a large scale multi-centered trial, collating quantified values with clinical course.

**Methods:**

In 638 subjects including 303 patients with HCC, 89 with chronic hepatitis (CH), 45 with liver cirrhosis (LC) and 201 healthy individuals, we quantified serum hTERTmRNA using the real-time RT-PCR. We examined its sensitivity and specificity in HCC diagnosis, clinical significance, ROC curve analysis in comparison with other tumor markers, and its correlations with the clinical parameters using Pearson relative test and multivariate analyses. Furthermore, we performed a prospective and comparative study to observe the change of biomarkers, including hTERTmRNA in HCC patients receiving anti-cancer therapies.

**Results:**

hTERTmRNA was demonstrated to be independently correlated with clinical parameters; tumor size and tumor differentiation (P < 0.001, each). The sensitivity/specificity of hTERTmRNA in HCC diagnosis showed 90.2%/85.4% for hTERT. hTERTmRNA proved to be superior to AFP, AFP-L3, and DCP in the diagnosis and underwent an indisputable change in response to therapy. The detection rate of small HCC by hTERTmRNA was superior to the other markers.

**Conclusions:**

hTERTmRNA is superior to conventional tumor markers in the diagnosis and recurrence of HCC at an early stage.

## Background

Since the discovery of circulating nucleic acids (CNAs) in plasma in 1948, many diagnostic applications have emerged. Recently, CNAs instead of a protein has appeared on this scene of practical diagnostic assay, suggesting that cell-free CNAs in the plasma/serum of cancer patients have characteristics of tumor-derived nucleic acids. In addition to DNA-derived from tumor cells [1-4], a recent development in this new field is the finding of tumor-related RNA in the plasma/serum of cancer patients [5]. These features include tyrosine kinase mRNA [6], telomerase components [7,8], the mRNAs that are encoded by different tumor-related genes [9-13], and viral mRNA [14]. In one study, two telomerase markers in breast cancer yielded 44% of positive rates [7]. Nevertheless, telomerase RNA seems to be a promising marker by the reason that it can be found even in the serum of patients with small, undifferentiated breast cancers without any metastatic lesions. Dasi et al. showed that circulating telomerase RNA is a sensitive marker, using real-time reverse transcription-PCR (RT-PCR) [8].

The telomerase catalytic subunit (hTERT) exerts important cellular functions, including telomere homeostasis, genetic stability, cell survival and perhaps differentiation [15-20]. hTERTmRNA in serum was detected in breast cancer but not in benign diseases, suggesting that hTERT is available for cancer diagnosis [4].

HCC ranks high among the most common and fatal malignancies associated with hepatitis B virus (HBV) and hepatitis C virus (HCV) infection [5]. Although HCC patients receive possible medical treatments such as transcatheter arterial chemoembolization/embolization (TACE/TAE), radiofrequency ablation (RFA), and surgery for primary tumors, intrahepatic and extrahepatic recurrence frequently limit patient's survival [6]. Although the modalities such as ultrasonography (US) and conventional tumor markers such as α-fetoprotein-L3 (AFP-L3) and DCP are widely used and important for HCC detection in clinical scenes [7], they still do not provide an entirely satisfactory solution to detect HCC at the early stage. Since HCC has been recently classified as a complex disease with a wide range of risk factors and many cellular signaling pathways have been reported to be involved in hepatocarcinogenesis, a novel biomarker for HCC is required [21]. We previously reported that measurement of serum hTERTmRNA by real-time RT-PCR method was sensitive in detection of tumor-derived hTERTmRNA even in the HCC patients whose AFP levels were low [9], and was also useful even for other malignancies such as non-small cell lung cancer, ovarian cancer, and gastric cancer [22-24]. In this large-scale study that includes follow-up cases, we focused on HCC of all malignancies and assessed the clinical significance of hTERTmRNA measurement in HCC diagnosis and monitored the clinical course.

## Methods

### Patients and Sample Collection

**Figure 1:**
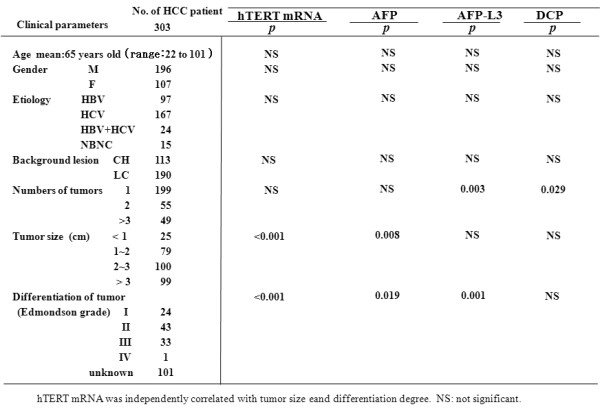
**Multivariate analysis of tumor markers with clinical parameters in patients with HCC**.

Four hundred-thirty seven consecutive patients (303 patients with HCC, 89 with CH, and 45 with LC), who were admitted at Tottori University related Hospitals, Osaka Red Cross Hospital, and Fukuoka University Chikushi Hospital between November, 2002 and December, 2006, were enrolled in this study. All the HCC patients had LC or CH as the underlying liver disease. The mean ages of patients with HCC, LC, and CH were 65, 66, and 61 years, respectively. One hundred-sixty seven patients were infected with HCV, 97 with HBV, 24 with both viruses and 15 with no viral markers. The patients were diagnosed by blood chemistry, US, computed tomography (CT), AFP and/or biopsy under US. The clinicopathological findings (age, gender, etiology, underlying liver disease (adjacent lesion), Pugh score, Child classification, total bilirubin (TB), albumin (Alb), alanine aminotransferase (ALT), AFP, AFP-L3, DCP, HCV titer, HCV subtype, tumor number, tumor size, differentiation degree of tumor, and presence of metastasis) were evaluated (Figure 1). HCC was diagnosed according to the AASLD guidelines and the differentiation of HCC was diagnosed by liver biopsy. Two hundred one healthy individuals including 144 females (from 24-87 years old: mean age 57 years) served as controls. Informed consent was obtained from each patient and the study protocols followed the ethical guidelines of the 1975 Declaration of Helsinki and were approved by the human research committee of Tottori University. The therapies for HCC include TAE, transcatheter arterial infusion (TAI), percutaneus ethanol injection therapy (PEIT), and RFA. Regarding follow-up patients, blood samples were taken basically every two months.

### RNA extraction and Real-time quantitative RT-PCR

Harvesting serum samples were performed as previously described [9]. RNA was extracted with DNase treatment from serum as reported previously [4,9]. The quantitative RT-PCR was performed as described previously [5,10]. (a) for hTERT. The RT-PCR condition was an initial incubation at 50 for 30 min followed by a 12-min incubation at 95, then 50 cycles at 95 (0 s), 55 (10 s), and 72 (15 s), and a 20 second melting at 40°C. The dynamic ranges of real-time PCR analysis for hTERTmRNA were more than approximately 5 copies in this assay and we were able to exclude the possibility of false negativity in serum samples from patients with CH, LC and controls. The PCR yielded products of 143 bp for hTERT (data not shown). The RT-PCR assay was repeated twice and the quantification was confirmed by using LightCycler (Roche, Basel, Switzerland) with reproducibility.

### hTERTmRNA during the treatment and detection of small HCC

We examined the therapeutic effectiveness of hTERTmRNA during the clinical course. Serum hTERTmRNA was measured before and 7 days after TAE in 16 HCC patients. In comparison with AFPmRNA, the half-life of hTERTmRNA was examined. By monitoring gene expression in serum up to 6 months after the beginning of therapy such as TAE, TAI, RFA, PEIT, surgical treatment, the effect of therapies were estimated in 20 patients. Furthermore, we examined hTERTmRNA expression and level of other conventional tumor markers after they were categorized by the tumor size (less than 10 mm, 11-20 mm, 21-30 mm, more than 30 mm).

### Immunohistochemistry

For immunohistochemical analysis, of 303 patients, 50 HCC patients (24 patients with HCV, 9 with HBV, 10 with both viruses, and 7 with unknown etiology; 5 patients with well-, 3 with well~moderately-, 32 with moderately-, 3 with moderately~poorly-, and 7 with poorly-differentiated HCC) with 35 positive and 15 negative conventional tumor markers, who underwent surgical treatment, were chosen. The immunohistochemical procedures were done as reported previously [25]. The sections were incubated with the following monoclonal antibodies: anti-hTERT (Santa Cruz Biotechnology, Santa Cruz, CA, USA), anti-Ki67 (Santa Cruz Biotechnology), anti-TUNEL (Sigma Chemical, MO, USA), HBsAg (Sigma Chemical), and HCV core antibody (Sigma Chemical). Expression degree was confirmed and estimated of hTERT, Ki-67, and TUNEL by the percentage of positively-stained cell number [26-28].

### Statistical analysis

Multivariate analysis was performed using SPSS 13.0 (SPSS Corp., Tokyo, Japan). Stratified categories in each clinical parameter were evaluated by One Way ANOVA and multivariate analysis using a logistic regression analysis model. To assess the accuracy of the diagnostic tests, the matched data sets (chronic liver diseases patients and HCC patients) regarding AFP, AFP-L3, DCP, and hTERTmRNA were analyzed by using receiver operator characteristic (ROC) curve analysis. The correlation of hTERTmRNA between HCC tissue and serum was analyzed using both Paired t test and Spearman's test. The detection rates of HCC in comparison with tumor size were evaluated by Friedman test.

## Results

### RNA extraction and Real-time quantitative RT-PCR

**Figure 2:**
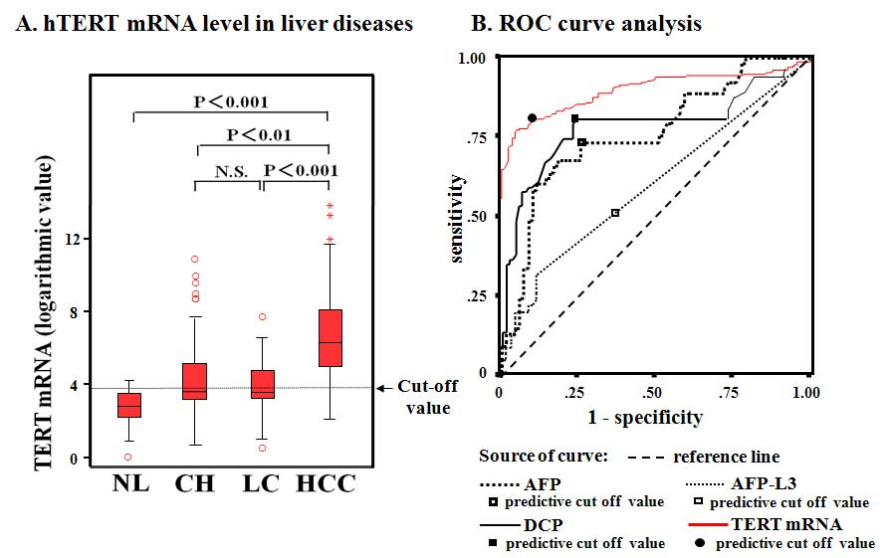
**Serum hTERTmRNA and AFP mRNA expression in patients with HCC, LC, and CH, and in healthy individuals and receiver operator characteristics (ROC) curve analyses of hTERTmRNA, AFP, AFP-L3, and DCP**. A. hTERTmRNA level in liver diseases. Serum hTERTmRNA levels and AFPmRNA level in patients with HCC, LC, CH, and healthy individuals by real-time RT-PCR were shown. The 95% confidence interval in each group is shown beside the dots. Significant differences between 4 groups are shown in the upper part of the figure. NL, individual with normal liver; CH, chronic hepatitis; LC, liver cirrhosis; HCC, hepatocellular carcinoma. Optimal cut-off values for hTERTmRNA expressions were predicted as 9,332 copies/0.2 ml. B. ROC curve analysis of hTERTmRNA, AFP, AFP-L3, and DCP was obtained by importing quantified raw data into SPSS II software. The data were analyzed by Paired t test (P = 0.01) and non-parametric Spearman's test (P = 0.017). AUC of each biomarker in ROC curve analysis is shown. Receiver operator characteristic (ROC) curve analysis of hTERTmRNA, AFP, AFP-L3, and DCP was obtained by importing quantified raw data into SPSS II. The solid line, bold dotted line, dotted line, and bold solid line correspond to DCP, AFP, AFP-L3 and hTERTmRNA, respectively.

In each quantitative assay, a strong linear relation was demonstrated between copy number and PCR cycles using RNA controls for concentration (r^2 ^> 0.99; data not shown). hTERTmRNA expression showed stepwise up-regulation with disease progression and the quantification was significantly higher in HCC than in LC, CH and healthy individuals (P < 0.001, P < 0.01 and P < 0.001, respectively, Figure 2A). ROC curve analyses showed that the sensitivity/specificity of hTERTmRNA for HCC were 90.2%/85.4% (Figure 2B). Optimal cut-off values for hTERTmRNA expressions were predicted as 9,332 copies/0.2 ml by stressing the higher specificity. Forty six (15%) of HCC patients, whose AFP, AFP-L3, and DCP were within normal limits, had 4.23 ± 0.32 logarithmic values of hTERTmRNA, and 20 patients of 46 patients were positive for this assay.

**Figure 3:**
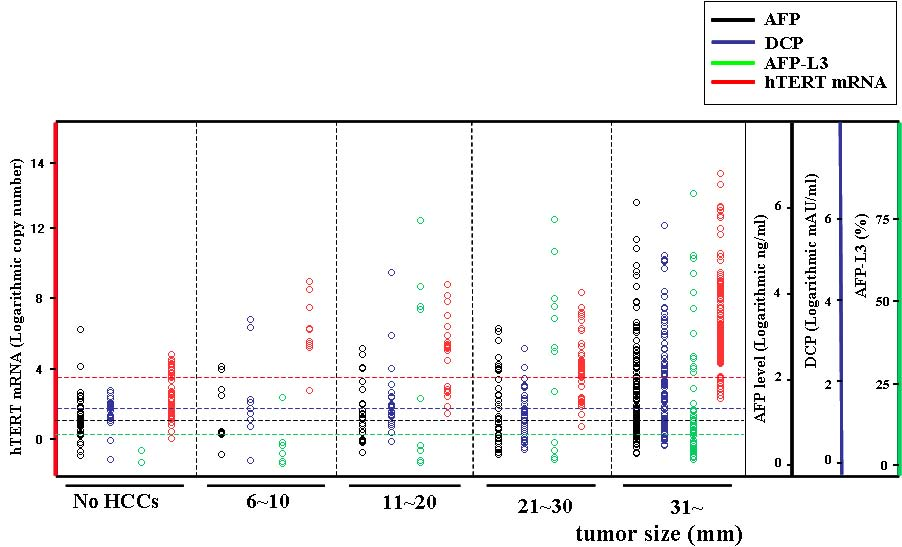
**Levels of hTERTmRNA in regard to tumor size (6-10 mm, 11-20 mm, 21-30 mm, and over 31 mm)**.

Multivariate analysis showed that hTERTmRNA was associated with tumor size and differentiation degree of tumor (P < 0.001, each, Figure 1 &3). However, hTERTmRNA was not associated with age, gender, etiology, background lesion or number of tumor. On the other hand, AFP was related to tumor size and differentiation (P = 0.008 and P = 0.0199), AFP-L3 was related to number of tumor and differentiation degree (P = 0.003 and P = 0.001), and DCP was associated with only number of tumor (P = 0.029). By Pearson relative test, serum hTERTmRNA significantly associated with tumor size and number of tumors (P < 0.033 and P < 0.003, respectively, Table 1). Importantly, hTERTmRNA was related only to DCP (P = 0.03).

**Table 1:** The sensitivity/specificity of each tumor marker for hepatocellular carcinoma and a statistic evaluation of hTERTmRNA level to clinical parameter were shown.

clinical parameter	**average ± S.E**.	Pearson test P value	Multivariate analysis P value
tumor size (mm)	21.2 ± 0.1	0.033	<0.001

(range: 6-90)			

tumor number	1.8 ± 0.1	0.003	N.S.

			

tumor differentiation		N.S.	<0.001

			

AFP (ng/ml)	6146 ± 4554	N.S.	N.S.

(n = 353)			

AFP-L3 (%)	6.7 ± 1.0	N.S.	N.S.

(n = 213)			

DCP (mAU/ml)	18780 ± 1044	0.03	N.S.

	n = 346)		

ROC curve analyses showed that the sensitivity/specificity of hTERTmRNA for HCC were 90.2%/85.4% (Table 2). The sensitivity/specificity of AFP, AFP-L3, and DCP were 76.6/66.2, 60.5/88.7, and 83.4/80.3, respectively. Thus, hTERTmRNA was superior to other markers especially in sensitivity. The positive predictive value (PPV)/negative predictive value (NPV) of hTERTmRNA were 83.0/85.9. On the other hand, the PPV/NPV for AFP, AFP-L3, and DCP were 74.6/67.7, 59.6/92.2, and 78.4/73.5, respectively. Consequently, hTERTmRNA was superior to other markers in the diagnosis of HCC. Combinations of hTERTmRNA with AFP level improved the sensitivity/specificity up to 96.0%/87.2%. ROC curve analysis categorized by viruses was examined and sensitivity/specificity in HBV-infected cases was similar to that of HCV-infected cases (additional file 1). hTERT and other markers in LC was not statistically and significantly different in comparison with that in CH.

**Table 2:** The sensitivity/specificity of each tumor marker for HCC was depicted.

	Sensitivity	Specificity	OR	PPV/NPV	Cut-off point
hTERTmRNA	90.2	85.4	19.0	83.0/85.9	3. 97 (logarithmic copy number)

AFP	76.6	66.2	11.1	74.6/67.7	<10 (ng/ml)

AFP-L3	60.5	88.7	2.2	59.6/92.2	<10 (%)

DCP	83.4	80.3	7.6	78.4/73.5	<40 (mAU/ml)

The sensitivity/specificity values are 90.2%/85.4% for hTERTmRNA, 76.6%/66.2% for AFP, 60.5%/88.7% for AFP-L3, and 83.4%/80.3% for DCP. Regarding a diagnostic assessment in sensitivity and specificity, hTERTmRNA is identified as the most excellent tumor marker. OR: odds ratio, PPV: positive predictive value (%), NPV: negative predictive value (%).

### Estimation of therapeutic effect and the possibility of early HCC detection of hTERTmRNA in comparison with other biomarkers

**Figure 4:**
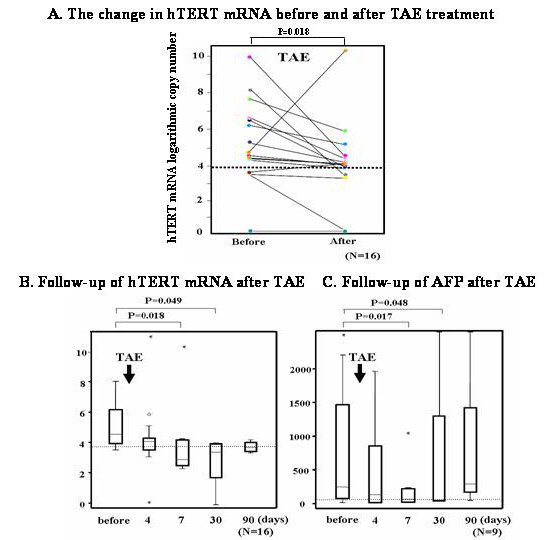
**The change of hTERTmRNA before and 7days after TAE**. A. Follow-up of serum hTERTmRNA before, 4, 7, 30 and 90 days after TAE. B. Follow-up of serum AFP before, 4, 7, 30 and 90 days after TAE.

To examine the significance of hTERTmRNA before and after TAE, serum hTERTmRNA was measured before and 7 days after TAE in 16 HCC patients (Figure 4A). As a result, hTERTmRNA significantly decreased after TAE (P = 0.018), suggesting that changes in hTERTmRNA are indicative of therapeutic effects on HCC. Comparing the follow-up data of hTERTmRNA and AFP (Figure 4B, C), the half-life of hTERTmRNA was shorter than that of AFP.

**Figure 5:**
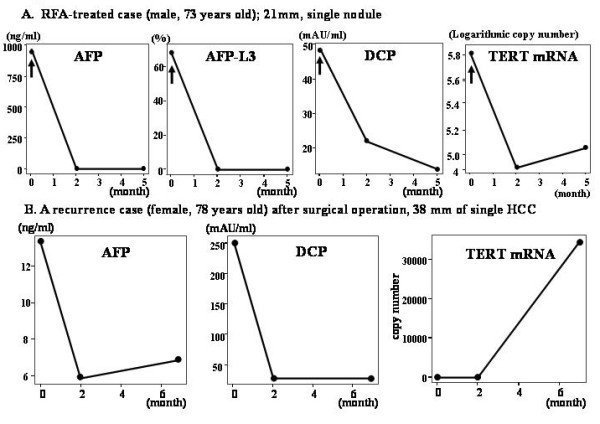
**Conventional tumor markers and hTERTmRNA detection during clinical course with therapeutic modalities**. A. Changes of AFP, AFP-L3, DCP and hTERTmRNA in RFA-treated case (male, 73 years old), of which HCC was 21 mm-sized and single. B.	Changes of AFP, DCP and hTERTmRNA in surgical operation-treated case (female, 78 years old), of which HCC was 38 mm-sized and single.

To clarify the significance of hTERTmRNA in monitoring the effect of therapies in comparison with other biomarkers, two representative cases were depicted in Figure 5. The quantification of hTERTmRNA was performed before, 2 and 5 months after RFA in a 73-year-old male patient whose HCC was a single 21 mm-sized (Figure 5A). hTERTmRNA changed similar to AFP, AFP-L3, and DCP, suggesting that hTERTmRNA is useful for monitoring the clinical course of HCC. In a 78-year-old female patient whose HCC was a single 38 mm-sized, a surgical operation was performed (Figure 5B). The values of AFP, DCP, and hTERTmRNA were measured before, 2 and 7 months after the operation. The operation was performed successfully in this patient, however recurrence was found by dynamic CT at 7 months after the operation. Although neither AFP nor DCP detected the recurrence, only hTERTmRNA did. In all the cases that hTERT detected recurrence in the earlier stage, no other imaging modality could detect it at the same time, but when we could find HCC in images such as US, CT, or MR, other markers began to arise.

**Figure 6:**
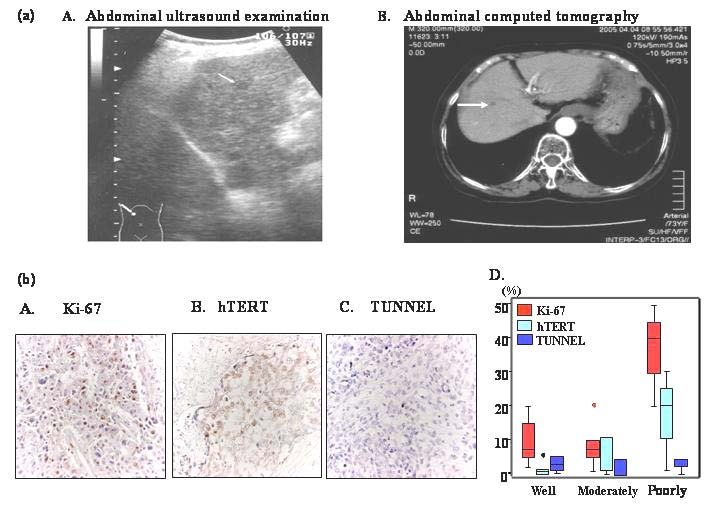
**Early detection of small HCC by circulating hTERT mRNA and immunohistochemical analysis of HCC tissues**. (a) Imaging diagnosis regarding case with 6 mm HCC. A. Ultrasonography and B. computed tomography of the smallest HCC detected by hTERTmRNA. The diameter of HCC was 6 mm. Left, ultrasonography; right, CT (b) Immunohistochemical analysis of HCC tissues. A. Ki-67 staining (×400), B. hTERT staining (×400), C. TUNEL (×400), D. Labeling indices of Ki-67, hTERT and TUNEL in regard to differential degree of HCC.

Finally, we examined the relationship between the positive rates of biomarkers and tumor size. Positive rate of hTERTmRNA was higher than that of the other markers in each category of tumor size; 6-10 mm, 11-20 mm, 21-30 mm, over 31 mm by Friedman test (P = 0.017) (Figure 3). However, the positivity of hTERTmRNA expression tended to reduce slightly in tumors with diameters that exceeded than 51 mm (5.2 ± 1.9 for 56 patients with 31-50 mm of HCC, 5.0 ± 1.8 for 43 patients with HCC over 51 mm; mean ± S.D.) (additional file 2). Dot blot regarding the correlation of hTERT mRNA quantification with tumor differentiation is shown in additional file 3. In a 6 mm HCC case, no marker other than hTERTmRNA was elevated and only abdominal US caught the evidence of HCC (Figure 6(a) A, B).

### Immunohistochemistry

Immunohistochemical analysis showed that Ki-67 positivity was observed in the nuclei of cancer cells (Figure 6(b) A). hTERT was observed in both the nuclei and cytoplasm of cancer cells (Figure 6(b) B). Some TUNEL-positive cells were present in cancerous lesions, however the prevalence was low (Figure 6(b) C). hTERT expression was significantly associated with the labeling index of Ki-67 (P = 0.023). When the labeling indices of Ki-67, hTERT and TUNEL were compared with the differentiation degree of HCC, both hTERT and Ki-67 were higher in poorly differentiated HCC than in well and moderately differentiated HCC (Figure 6(b) D).

## Discussion

Since HCC has been recently classified as a complex disease with a wide range of risk factors and many cellular signaling pathways have been reported to be involved in hepatocarcinogenesis, a novel biomarker for HCC is required [21]. Since an epoch-making assay to detect telomerase activity was established [11], telomerase has been examined in many kinds of cancers, precancerous lesions and normal tissues using the telomeric repeat amplification protocol and investigated the correlation with telomere length [29,30]. Notwithstanding that telomerase was definitely an unprecedented candidate tumor marker due to its specificity to cancer, it has clinically remained inapplicable because telomerase expression has not been detected stably in body fluid [12]. In serum, the hTERTmRNA derived from cancer cells seemed to be undetectable because it becomes instable by RNase in blood. Since RNAs in serum are unexpectedly stable within 24 hrs after drawing blood due to particle-associated complex in structure [13,14], it has been suggested that they can be generally detected even in RNase-rich blood. Actually, hTERTmRNA can be detected in serum from breast cancer patients and its maximum sensitivity and specificity are at most 40% and 100%, respectively [4]. The sensitivity in patients with HCC rose to 89.7% in the semi-quantitative assay, and thus compared favorably with the previous findings in which the sensitivity and specificity of AFPmRNA were 69% and 50% for HCC, respectively [31]. Besides, with respect to HCC detection, AFPmRNA was superior to AFP level used routinely in clinic [32]. Recently, in the present study, we reported the sensitivity to detect the nucleotides in blood in the process of RNA extraction, including centrifugation steps less than 1500 × g to remove cellular proteins in serum and a primer set that can detect hTERTmRNA more efficiently than primers in the previous reports (data not shown). We previously reported that hTERT expression was very faint in the serum from normal individuals indicating that lymphocytes and circulating normal cells express very low levels of hTERTmRNA [9]. Because hTERTmRNA in lymphocytes is very low, elevated hTERTmRNA levels in serum may mean that hTERTmRNA is derived from cancer cells. Since we could detect negligible amounts of lymphocyte markers after three steps of centrifugation of blood samples, the RNA extraction procedure seemed to remove lymphocytes effectively. In addition, normal or damaged hepatocytes express negligible amounts of hTERT [33,34]. Furthermore, we previously showed the significant correlation of hTERTmRNA expression between tumor tissue and serum [32]. These data suggest that hTERTmRNA detected in serum is derived from tumor cells.

Previously, we reported that qualitative analysis of serum hTERTmRNA was superior to AFP for the purpose of the early detection of HCC, because hTERTmRNA was detectable in HCC patients with normal AFP levels [9]. AFP is being widely used as a reliable marker of HCC not in earlier stage but in the advanced stage [35]. However, in this study, neither AFP was able to distinguish HCC from non-cancerous liver diseases, nor hTERTmRNA was correlated with AFP level (P = 0.201), suggesting that quantitative analysis of serum hTERTmRNA was much more sensitive for HCC diagnosis even in the early stage. Because the induction of the abdominal (enhanced-)US, CT, and MRI into the clinical scene enabled us to detect smaller-sized HCC [36], the sensitivity of AFP in the early detection of HCC became less than 70%. Unlike AFP level, AFPmRNA was significantly correlated with hTERTmRNA (P < 0.001) and more sensitive than AFP. In the present study, we measured AFP-L3, since AFP-L3 has been reported to be a more HCC-specific marker than AFP [37]. Indeed, the level of AFP-L3 correlated significantly with differentiation and number of HCC although that of AFP was correlated with tumor size and differentiation.

In the present study, of 303 HCC patients, 24 patients were negative below the calculated cut-off value (9,332; 3.97 as logarithmic number) for serum hTERTmRNA. Although the reason why hTERTmRNA was negative in these patients is not clear, eleven of 24 hTERTmRNA-negative HCC patients had decompensated liver cirrhosis as the underlying disease. It has been reported that decompensated liver cirrhosis had higher levels of serum TGF-β that promotes apoptosis of immortalized hepatocytes and, in these cases, elevated TGF-β may stimulate apoptosis, resulting in reduction of hTERTmRNA [34,38,39]. hTERT-negative cases had no other common characteristics with age, gender, etiology, child classification etc. than tumor size, ALT, and surrounding lesion. In 23 cases (95.8%), ALT was within 1.5 fold normal limits. In 17 cases (70.8%), surrounding lesion was LC including decompensated situation. Tumor size in 12 cases (50%) was over 30 mm, reflecting on the biological features of cancer itself, as referred in Norton-Simon models regard tumor growth [40]. AFP and DCP were positive in 16 (66.7%) and 11 (45.8%) cases, respectively, suggesting that combinative use of these markers contributes to improve the diagnostic specificity.

Thus, hTERTmRNA is not only improved in both sensitivity and specificity but has a close correlation with tumor size and number in an early stage of HCC. Since HCC repeatedly recurs polyclonally after any treatment as a biological characteristic, the measurement of serum hTERTmRNA makes it possible to recognize recurrence or therapeutic effect in details as well as the usefulness for one-point diagnosis. In this respect, we have to undergo follow-up study after the treatment of HCC [24]. hTERTmRNA expression was closely associated with well to moderate differentiation degree of HCC and was enhanced with the proliferation. We should clarify that serum hTERTmRNA can be detected by what alterations of other molecules during the cancer progression [41-43]. In lower differentiated HCC, tumor cells are proliferating and hTERTmRNA has a tendency to correlate with the differentiation degree and an apoptotic event never reflect on the serum detection of cancer cell-derived mRNAs (Figure 6). Nakashio et al. previously reported the significant correlation of HCC differentiation with telomerase expression [44]. The results in the present study confirmed their findings. hTERTmRNA showed more sensitivity and specificity compared with AFPmRNA in HCC patients. However, in liver diseases other than HCC, hTERTmRNA was not correlated with AFPmRNA. The higher specificity of hTERTmRNA in HCC may be related to fact that AFPmRNA is produced in HCC cells and injured hepatocytes and hTERT is produced mainly in HCC cells. Furthermore, we could detect serum hTERTmRNA expression even in HCC patients with less than 10 mm moderate-differentiated tumor, indicating that hTERT are upregulated during rapid proliferation of tumor at the early phase of oncogenesis, de-differentiation.

Waguri et al. proved that there exist circulating cancer cells derived from original HCC tissues in blood and they can detect hTERTmRNA in blood [45]. The present study suggests that quantification of hTERTmRNAs in serum has diagnostic implications for HCC. Unless apoptosis of cancer cells contributes to the early detection of HCC using serum mRNA, the essence may be immunoreactions [46]. The development of micro vessels may be also involved in the step [47]. We will evaluate the correlation of prognosis with hTERTmRNA and the availability of hTERTmRNA in other cancers by comparison of hTERTmRNA with other tumor markers [48], and will study its usefulness for inflammatory diseases in which cellular reactions are active [49]. This method depends on RNA stability in each process of RNA purification, storage, and quantification. In the light of its superior positivity to other markers, the assay will be applied for clinical use in the strict condition because it is required to keep the serum RNA as it is in blood and avoid the degradation of RNA quality. Now we are improving RNA stability and PCR condition to better cost/benefit of this assay. In the future, another large-scale study will be required to confirm our results for monitoring HCC and the feasibility for its detection even on a primary care level.

## Conclusions

In sum, our results support the suggestion that quantification of circulating hTERTmRNA expression is clinically useful for the early detection of HCC. Furthermore, hTERTmRNA is superior to conventional tumor markers in the diagnosis and recurrence of HCC at the early stage.

## Abbreviations

(h)TERT: (human) telomerase reverse transcriptase protein; HCC: hepatocellular carcinoma; HCV: hepatitis C virus; HBV: hepatitis B virus; LC: liver cirrhosis; CH: chronic hepatitis; AFP: α-fetoprotein; DCP: des-γ-carboxy prothrombin; ALT: alanine aminotransferase; Alb: albumin; CNA: Circulating nucleic acids.

## Competing interests

The authors declare that they have no competing interests.

## Authors' contributions

YO analyzed biomedical data and provided blood sample as main researcher in Osaka Red Cross Hospital. MN analyzed biomedical data in Kinki University. MK analyzed biomedical data and provided blood sample as main researcher in Kinki University. KY analyzed biomedical data and provided blood sample in San-in Labor Welfare Hospital. TK analyzed clinical data and the practical analysis in San-in Labor Welfare Hospital. KO analyzed HCC imaging data and the analysis in Saiseikai Gotsu General Hospital. YK was in charge for case study in Saiseikai Gotsu General Hospital. SM analyzed biomedical data and provided blood sample as main researcher in Saiseikai Gotsu General Hospital EN was in charge for case study and biomedical analysis in Saiseikai Gotsu General Hospital. YH analyzed clinical and biomedical data as main researcher in Saiseikai Gotsu General Hospital comprehensively. MK analyzed biomedical data and provided blood sample as main researcher in Matsue City Hospital. SS analyzed biomedical data and provided blood sample as main researcher in Fukuoka University Chikushi Hospital. YH performed biomedical and clinical analysis in surgical case in Tottori University. HK analyzed biomedical data and provided blood sample as chief researcher in San-in Labor Welfare Hospital. JH provided the environment to analyze the data comprehensively. All authors read and approved the final manuscript.

## Pre-publication history

The pre-publication history for this paper can be accessed here:

http://www.biomedcentral.com/1471-230X/10/46/prepub

## Supplementary Material

Additional file 1 **TIF ROC curve analysis and AUC in measurement categorized by viruses**. ROC curve analysis and AUC in measurement categorized by viruses are demonstrated. Sensitivity/specificity of hTERTmRNA expression in HBV-infected cases is similar to that in HCV-infected cases. Click here for file

Additional file 2 **MS word Positivity of each marker for HCC**. Positivity of each marker for HCC was shown, categorized by tumor size. Click here for file

Additional file 3 **TIF Dot blot regarding the correlation of hTERTmRNA quantification with tumor differentiation**. Serum hTERTmRNA quantification in HCC patients (n = 101) diagnosed by liver biopsy was shown, categorized by tumor differentiation. The quantification in serum of HCC patients with well-/moderately-/poorly-/un-differentiation was 4.4 ± 1.4/5.4 ± 2.0/6.3 ± 3.3/5.9 ± 1.8 (mean ± SD). Click here for file
