# Supplementary figures and images for: A novel biomarker TERTmRNA is applicable for early detection of hepatoma
Source: BMC Gastroenterol. 2010 May 18;10:46. doi: 10.1186/1471-230X-10-46 (PMC2881114; doi:10.1186/1471-230X-10-46)

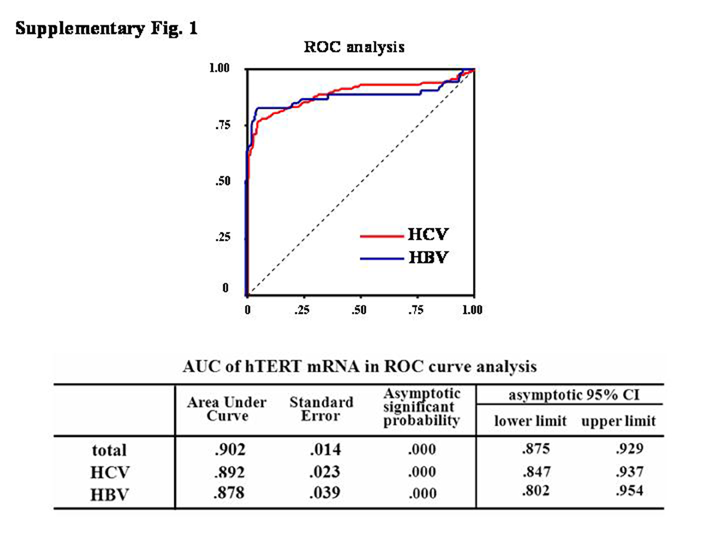

Supplement: Additional file 1 — TIF ROC curve analysis and AUC in measurement categorized by viruses. ROC curve analysis and AUC in measurement categorized by viruses are demonstrated. Sensitivity/specificity of hTERTmRNA expression in HBV-infected cases is similar to that in HCV-infected cases. [file 1471-230X-10-46-S1.TIFF]

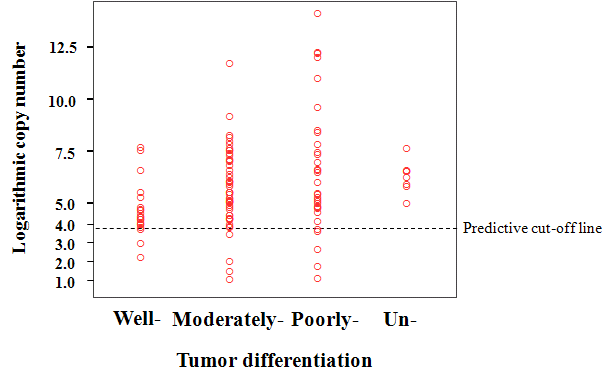

Supplement: Additional file 3 — TIF Dot blot regarding the correlation of hTERTmRNA quantification with tumor differentiation. Serum hTERTmRNA quantification in HCC patients (n = 101) diagnosed by liver biopsy was shown, categorized by tumor differentiation. The quantification in serum of HCC patients with well-/moderately-/poorly-/un-differentiation was 4.4 ± 1.4/5.4 ± 2.0/6.3 ± 3.3/5.9 ± 1.8 (mean ± SD). [file 1471-230X-10-46-S3.TIFF]
