# Supplementary material for: A novel biomarker TERTmRNA is applicable for early detection of hepatoma
Source: BMC Gastroenterol. 2010 May 18;10:46. doi: 10.1186/1471-230X-10-46 (PMC2881114; doi:10.1186/1471-230X-10-46)
Supplement: Additional file 2 — MS word Positivity of each marker for HCC. Positivity of each marker for HCC was shown, categorized by tumor size. [file 1471-230X-10-46-S2.DOC]

Supplementary Table 1
